# Host–Parasite Relationship—Nematode Communities in Populations of Small Mammals

**DOI:** 10.3390/ani12192617

**Published:** 2022-09-29

**Authors:** Milan Miljević, Borislav Čabrilo, Ivana Budinski, Marija Rajičić, Branka Bajić, Olivera Bjelić-Čabrilo, Jelena Blagojević

**Affiliations:** 1 Department of Genetic Research, Institute for Biological Research “Siniša Stanković”—National Institute of the Republic of Serbia, University of Belgrade, Bulevar Despota Stefana 142, 11000 Belgrade, Serbia; 2 Department of Biology and Ecology, Faculty of Sciences, University of Novi Sad, Trg Dositeja Obradovića 2, 21000 Novi Sad, Serbia

**Keywords:** rodent, spleen mass, body condition, parasite species richness, parasite load

## Abstract

**Simple Summary:**

Intestinal nematodes were analyzed in three small rodent species: *Apodemus sylvaticus*, *Apodemus flavicollis*, and *Myodes glareolus.* In total, 15 species of nematodes were identified. We studied the relationship between the parasitological parameters (individual parasite species richness IndPSR and individual parasite load IndPL) and morphological characteristics (body mass, body condition index BCI, and spleen mass). In all of the studied species, our results showed that animals in a better condition (BCI) had significantly higher parasite species richness, as we had expected. Furthermore, we showed that females of the same BCI as males were parasitized with more parasite species in *A. flavicollis*. Contrary to our expectations, the spleen mass did not reflect changes in the level of parasitism, but in *M. glareolus*, a smaller spleen was related to a higher parasite load. Since the spleen is just part of the complex immune response, it is possible that the presence of nematode parasite species provokes, in small rodent species, a response at the other levels of the immune system.

**Abstract:**

Nematode burdens and variation in morphological characteristics were assessed in eighty-eight animals from three host species (*Apodemus sylvaticus*, *Apodemus flavicollis*, and *Myodes glareolus*) from eight localities in Serbia. In total, 15 species of nematodes were identified, and the overall mean parasite species richness (IndPSR) was 1.61 per animal (1.98 in *A. flavicollis*, 1.43 in *M. glareolus*, and 0.83 in *A. sylvaticus*). Furthermore, the studied host species significantly differed in individual parasite load (IndPL) and in the following morphological characters: spleen mass, body condition index (BCI), and body mass. We aimed to analyze the relationship between the burden of intestinal nematodes, on one hand, and the body conditions of the host and its capability to develop immune defends on the other. Spleen mass was considered as a measure of immune response. In all host species, larger animals with a better condition (higher BCI) were infected with more parasites species (IndPSR), while parasite load was not related to BCI. Only in *A. flavicollis* were males significantly larger, but females of the same sizes were infected with more parasite species. This female-biased parasitism is contrary to the theoretical expectation that males should be more parasitized, being larger, more active, with a wider home range. Although the spleen size was significantly correlated with body condition and body mass, IndPSR was not related to spleen mass in any studied species, but in *M. galareolus*, we found that a smaller spleen was related to higher infection intensity (IndPL).

## 1. Introduction

Mammals can be infected with various parasites, from viruses and bacteria to macroscopic worms and arthropods. In wild populations, different intrinsic and extrinsic factors affect the variation in parasite burdens. Population density and the species geographic range could considerably affect the parasite diversity [1]. Host species with a small geographical range and low population density often display a lower diversity of parasite species [2]. Morand and Poulin’s [3] results suggest that opportunities for parasite colonization depend more closely on how many hosts are available in a given area than on how large the hosts are.

Some external or internal characteristics of the host may be related to the presence of parasites. Thus, mammalian body mass is not a determinant of parasite species richness, which means that increases in body mass do not lead to increases in parasite species richness [3,4,5,6,7,8]. However, a positive relationship between parasite richness and host body mass has been shown for various terrestrial mammal species [9,10,11]. Island biogeography theory describes hosts as “island habitats” for their parasites, and larger hosts have often been predicted to harbor richer parasite faunas. It has been considered that larger body hosts provide larger habitat patches and a greater variety of niches [3,6,8,12].

While body mass is a physical quantity obtained by measurement, body condition index is based on the ratio of the body mass/body length. Since body mass influences the body condition, a question can be raised about the relationship of this parameter with infection intensity. Body condition is treated as a proxy of animal health [13] or as a measure of individual energetic status [14,15], which significantly impacts reproductive success [16]. Furthermore, body condition may be related to infection intensity [13,17], but may also depend on the host sex, locality, and life history aspects [15,18]. Environmental factors could influence the relationship between the infection and the body condition of the host in different ways. For example, lower food availability can lead to home range expansion [19], thus increasing the possibility of infection [20]. As a result, a negative correlation between the body condition and infection intensity can occur.

In the context of spatial distribution, another important factor is the difference in the home range size between males and females. Radiotracking results showed that males of the yellow-necked mouse *Apodemus flavicollis* (Melchior, 1834) had a wider home range than females [19], so a higher risk of infection for them is expected. In most vertebrates, males are more heavily parasitized than females [21,22]. Sex-biased parasitism is often seen as a consequence of differential investments in immune function and immunosuppression caused by androgens in males [13,23,24]. Altogether, the social organization of the species and an organism’s ecology significantly impact the immune system and protection against pathogens.

Among the mammals, rodents are particularly suitable for studies of intestinal helminths since they are abundant in nature, relatively easy to trap, and are often found near human settlements. Nematodes are commonly the dominant group of helminths in rodents [18,25,26,27], and this can be related to their diet. The wood mouse *Apodemus sylvaticus* (Linnaeus, 1758) and *A. flavicollis* are typically granivorous [28] and thus have limited opportunities to ingest infective stages of digeneans and cestodes. There is a rich body of literature on the helminth fauna of rodents in Europe, most of which is focused on *A. sylvaticus* [18,25,26,29,30]. The helminth fauna of other rodent species including *A. flavicollis* and the bank vole *Myodes* (=*Clethrionomys*) *glareolus* (Schreber, 1780) has also received attention in various European countries [27,31,32,33,34].

Research on the nematode parasite diversity of small mammals in Serbia has increased in the last decade. Bjelić-Čabrilo et al. [35] conducted extensive research by describing helminth fauna’s quantitative and qualitative composition in a sample of 588 bank voles. Extensive parasitological studies of the yellow-necked and wood mouse were also carried out [36,37,38,39]. Furthermore, studies of the intestinal nematode community’s interaction with immune response and gene expression were conducted on the yellow-necked mouse [40]. In general, research dealing with the effect of parasites on the immune response and body condition of their rodent hosts is relatively rare [18,41,42,43].

The spleen is an organ with a vital role in immune defense, and its size (length or mass) is often used as an indicator of immune activity in birds and mammals [18,41,42,44,45,46]. It is assumed that a larger spleen produces and stores more lymphocytes [47]. When the spleen mass reflects immune-competence, a positive correlation is expected to the parasite load or parasite species richness at the interspecies level [42,48]. Specifically, at the intraspecies level, positive correlation between the spleen mass and parasite load represents a measure of immune response as a physiological reaction to parasites present in the host organism [45,49]. The number of nematode species exploiting a host species (nematode species richness) may better explain the selective pressure than nematode prevalence [48].

Here, we describe the diversity of intestinal nematode fauna and parasite burden in the wood mouse, yellow-necked mouse, and bank vole by calculating the individual parasite species’ richness (IndPSR) and individual parasite load (IndPL). Additionally, our objectives were to investigate the relationships between measures of immune response (spleen mass), intrinsic factors such as body mass and body condition, and calculated parasitological parameters (IndPSR and IndPL). We expected an increase in IndPSR and/or IndPL with the increase in body condition index (BCI). Regarding gender differences in BCI, we expected the gender with a higher BCI to be more parasitized. Furthermore, we expected increased parasitism to positively correlate with spleen mass as the measure of immune defense.

## 2. Materials and Methods

A total of 88 rodents of three species were trapped at eight different localities in Serbia: wood mouse, *A. sylvaticus* (eight females and 10 males); yellow-necked mouse, *A. flavicolis* (23 females and 26 males) and bank voles, *M. glareolus* (eight females and 13 males). All individuals of *A. sylvaticus* were caught in Belgrade’s urban forest (Zvezdarska šuma), while *A. flavicollis* was collected from seven and *M. glareolus* from four localities in Serbia (Table 1).

The animals were trapped from March to October 2014 to 2018 using Longworth live traps, which contained dry hay and wheat grain placed in the nest box, baited with a mixture of oat flakes and sardines. Traps set in the afternoon were checked in the early morning, and caught animals were transported to the laboratory in appropriate cages. Morphometric data such as body mass (g) and body length (mm) were measured for all of the captured animals. The spleens were removed, and mass weighed in milligrams (mg) with a precision of ±0.1 mg. The host body condition or body condition index (BCI) was calculated as the body mass/body length ratio [18]. In the sample, there were no pregnant females. Animals were treated according to the legal and ethical guidelines as indicated in Directive 2010/63/ EU of the European Parliament and Council of 22 September 2010 on the protection of animals used for scientific purposes. The Ethics Committee of the Institute for Biological Research, “Siniša Stanković,” National Institute of the Republic of Serbia, approved the research protocol.

### 2.1. Parasitological Techniques

Part of the intestinal tract (stomach, small intestine, caecum, colon, and rectum) was removed from each animal and cut longitudinally. Intestinal contents were released into a Petri dish and then into a larger conical glass with water. The supernatant was cast until the content was clear. Collected parasites were conserved in 70% ethanol and determined morphologically, according to Ryžikov [50,51] and Genov [52].

Two metrics were used: individual parasite load IndPL, which described the total number of all intestinal nematodes of all species in each host, and individual parasite species richness IndPSR, or the total number of intestinal nematode species encountered in each animal [18]. Parasitological quantitative parameters (prevalence, mean infection intensity, and mean abundance) were calculated according to Bush et al. [53].

### 2.2. Statistical Approach

Statistical analyses were performed using Statistica 12 software (StatSoft, V12, Tulsa, OK, USA, 2013), and the significance level was defined as *p* < 0.05. The set of morphological traits (body mass, spleen mass, BCI) and two parasitological traits were tested for normality. Non-normal variables were log transformed. The Kruskal–Wallis test was performed for the analyses of parasite species richness among the studied hosts. Differences between males and females in spleen mass, IndPSR, and IndPL were tested using the Mann–Whitney U tests. Differences in the body mass and body condition index were tested using one-way ANOVA.

We performed regression analyses to study the relationships between the parasitological parameters (IndPSR, IndPL) and morphological traits (BCI, spleen mass). The slope of the regression lines was compared using a *t*-test.

## 3. Results

### 3.1. Parasites Diversity and Parasite Loads

A total of 15 nematode species were recorded from the three host species (Table 2). The highest number of identified species (12) was recorded in *A. flavicollis*. The number of intestinal nematode species varied from two at the locality Ruski Krstur to nine at Vlasina. Host populations of *A. flavicollis* and *M. glareolus* had a very high overall prevalence of infection (100%), while the infection prevalence of *Apodemus sylvaticus* was 55.5%. Only two nematode species, *Heligmosomoides polygyrus* and *Aonchotheca annulosa*, were present in all three of the studied host species with differing prevalence. The most prevalent parasite in both the *Apodemus* host species was *H. polygyrus*, while in the bank vole, it was *Heligmosomoides glareoli* (Table 2).

Six nematode species (*Aspiculuris tetraptera*, *Eucoleus* sp., *Heterakis spumosa*, *Mastophorus muris*, *Rictularia proni*, *Syphacia stroma*) were only found in the yellow-necked mouse, while the species *Aonchotheca murissylvatici* and *Heligmosomum costellatum* were found only in the bank vole. The most abundant intestinal nematode in *A. flavicollis* was *Syphacia stroma* (702 individuals), while in *A. sylvaticus*, it was *Syphacia frederici* (369 individuals). *Aonchotheca murissylvatici*, with 70 individuals, was the most abundant species in *M. glareolus*. The number of parasite individuals within each host ranged from 1 to a maximum of 495 in a single yellow-necked mouse. Mean parasite species richness was the highest for *A. flavicollis* (1.98 ± 0.857), lower for *M. glareolus* (1.429 ± 0.746), and the lowest for *A. sylvaticus* (0.833 ± 0.857). When we excluded non-parasitized individuals, the mean parasite species richness in *A. sylvaticus* was 1.500 ± 0.527. Based on the Kruskal–Wallis test (H_(2,*n*=80)_ = 6.346, *p* = 0.042), differences in the mean parasite species richness were significant for the studied host species, although there were no significant differences between the pairs of host species.

### 3.2. Relationships between Parasite Load and Morphological Traits

In *A. flavicollis*, the males showed significantly higher body mass (F_(1,47)_ = 6.520, *p* = 0.0140) and BCI (F_(1,47)_ = 4.150, *p* = 0.047) than the females, while in *A. sylvaticus* and *M. glareolus*, sexual dimorphism did not vary for either parameter. Furthermore, no difference between the males and females was found for the spleen mass, IndPSR, or IndPL for the three studied host species (Mann–Whitney U tests, all *p* > 0.10).

Using regression analyses, we found that the spleen mass was significantly positively correlated with both body condition (*A. sylvaticus*: r = 0.630, *p* = 0.005; *A. flavicollis*: r = 0.586, *p* < 0.005; *M. glareolus*: r = 0.726, *p* = 0.003), and body mass (*A. sylvaticus*: r = 0.768, *p* < 0.001; *A. flavicollis*: r = 0.591, *p* < 0.001; *M. glareolus*: r = 0.740, *p* = 0.003). Correlations between body mass and BCI were highly significant (*p* < 0.001) in all of the studied species (*A. sylvaticus* r = 0.96; *A. flavicollis* r = 0.98; *M. glareolus* r = 0.98). Since the difference in all of them was <5%, we excluded body mass from further analyses.

#### 3.2.1. Body Condition Index (BCI) vs. Parasitological Parameters

**Figure 1:**
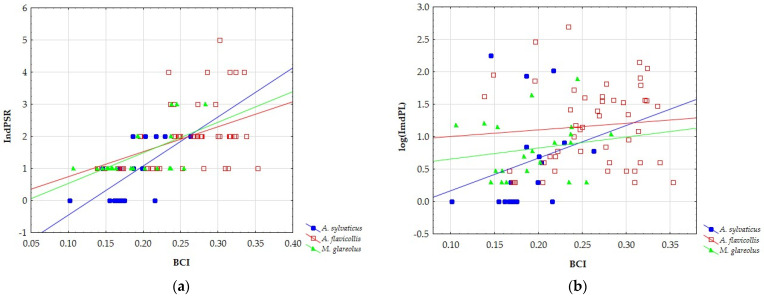
The relationship between the body condition index (BCI) to (**a**) individual parasite species richness IndPSR and (**b**) parasite load (IndPL).

IndPSR was significantly positively correlated with body condition for all of the studied rodent species: *A. sylvaticus* (r = 0.641; *p* = 0.004); *A. flavicollis* (r = 0.416; *p* = 0.003); *M. glareolus* (r = 0.588; *p* = 0.005) (Figure 1a). In contrast, correlations between IndPL and BCI in all host species were positive but non-significant: *A. sylvaticus* (r = 0.239, *p* = 0.340), *A. flavicollis* (r = 0.088; *p* = 0.548), *M. glareolus* (r = 0.170, *p* = 0.461) (Figure 1b).

**Figure 2:**
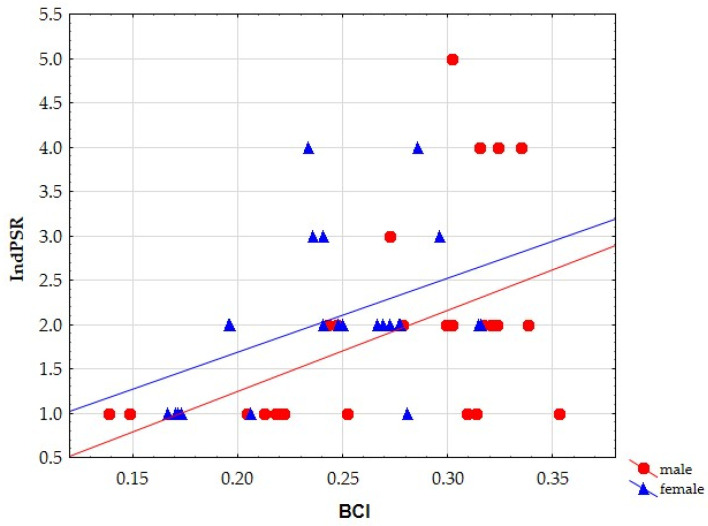
The relationship between individual parasite species richness (IndPSR) and body condition index (BCI) in males and females of the yellow-necked mouse *A. flavicollis.*

As the body mass and BCI in *A. flavicollis* differed significantly between the sexes, regression analysis was performed separately for this species. Both sexes showed significant positive increase in IndPSR with an increase in BCI (male: r = 0.457, *p* = 0.019; female: r = 0.443, *p* = 0.034). However, the regression line in females shifted up on the Y axis (Figure 2). Comparison of the regression slopes via the *t*-test showed significant differences (t = 2.872, df = 45, *p* = 0.006). The comparison of the regression slopes of IndPL on BCI showed no significant differences (t = 1.858, df = 45, *p* = 0.070) between sexes.

#### 3.2.2. Spleen Mass vs. Parasitological Parameters

**Figure 3:**
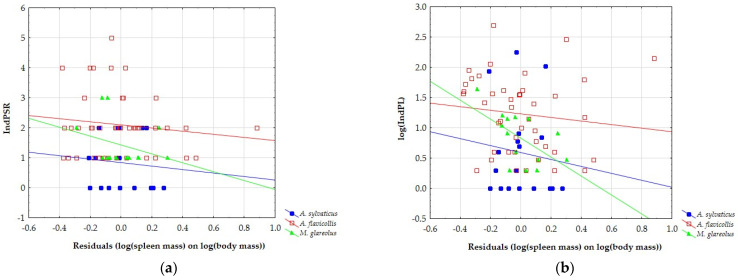
The relationship of spleen mass with (**a**) individual parasite species richness IndPSR and (**b**) individual parasite load (IndPL).

Since spleen mass was significantly positively correlated to body mass, which means that body mass explains a large part of the variance of the spleen mass, for further comparisons, we used spleen mass residuals (log(spleen mass) on log(body mass)). IndPSR showed no significant relation to the spleen mass (Figure 3a) in any of three host species in our study: *A. sylvaticus* (r = −0.099, *p* = 0.695); *A. flavicollis* (r = −0.134, *p* = 0.391); and *M. glareolus* (r = −0.307, *p* = 0.286). On the other hand, negative correlations between IndPL and spleen mass were non-significant in *A. sylvaticus* (r = −0.111, *p* = 0.660) and *A. flavicollis* (r = −0.123, *p* = 0.431), while in *M. glareolus*, it was significant (r = −0.579, *p* = 0.030; Figure 3b).

## 4. Discussion

It is estimated that there are more than 25,000 parasitic nematode species in vertebrates, most of which are still unidentified [54]. Although a small fraction of all nematode species are parasites, they have attracted the most attention in research. Our study showed that intestinal nematodes were highly prevalent in the studied species. Thus, they were present in all specimens of *A. flavicollis* and *M. glareolus*. *Heligmosomoides polygyrus* was the most prevalent parasite species of *A. flavicollis* (69.4%) and *A. sylvaticus* (33.3%). An identical value of total nematode prevalence (100%) was recorded for yellow-necked mice sampled across three different habitat types at one location in Croatia by Bužan et al. [34]. The authors also noted *H. polygyrus* as the most prevalent nematode species (66.7%). Adnađević et al. [40] registered a very high (96.7%) overall prevalence of nematodes in yellow-necked mice in Serbia, and the highest prevalence was detected for *H. polygyrus*. In our study, 15 intestinal nematodes were found in all host species, of which 12 nematode species were even identified in populations of the yellow-necked mouse from seven localities. The diversity of nematode parasite species was the highest in *A. flavicollis*, which was the largest species with the broadest home range in the current study. Čabrilo et al. [37] reported six nematode species from *A. flavicollis* at three mountainous localities in the Peripannonic region of Serbia. In a more extensive study, Čabrilo [55] identified nine nematode species from 305 specimens of the yellow-necked mouse from 18 different localities in Serbia, south of the Sava and Danube Rivers. Due to faster and more precise analysis, we intend to apply molecular methods to the determination of nematode species in future research.

All specimens of *A. sylvaticus* were collected in an urban forest Zvezdarska šuma, and the number of detected nematode species was only four. Similarly, Milazzo et al. [56] found four helminth species in populations of *A. sylvaticus* from the riverbed and bank section, but only two were found on the slopes’+ habitat in the studied location in Italy. A greater number of nine helminth species were detected in two areas in Spain in a sample of 917 specimens of *A. sylvaticus*, but these studies covered an 18-year period [57]. Our samples were neither spatially nor seasonally balanced among the analyzed species, so nematode diversity illustrates the current situation at the studied localities. All samples were collected in the same manner, in the woods, but comparisons among species that included a different number of locations per species did not reflect the actual situation in the territory of Serbia regarding the parasite species’ richness.

Our study showed that better-performing animals, with higher BCI, were infected with significantly more nematode species. Although males were larger than females in all studied species, their BCI only differed significantly between sexes in *A. flavicollis*. In this species, both sexes showed a significant positive correlation of IndPSR with body condition, but females with the same BCI as males were parasitized with more nematode species. There are a few possible reasons for this.

The first could come from the fact that females of *A. flavicollis* are characterized by multiple mating with different males [58]. The evolution of female promiscuity increases the chance for multi-parasite infection, but on the other hand, it assures fertilization and gives them a chance to increase the genetic quality of the offspring [59]. Multiple paternities were also found in natural populations of *A. sylvaticus* [60,61], *Clethrionomys glareolus* [62], *Apodemus agrarius* [60], *Apodemus microps* [63], and other small mammals. A second explanation for the higher infestation of *A. flavicollis* females could be that they nurse their offspring solitarily, as many rodent species do [64,65,66]. Thus, such nests could be suitable for parasite maintenance as hypothesized for *A. flavicollis* ectoparasites by Kowalski et al. [22]. Similarly, Bordes et al. [18] found an association between body condition and individual parasite species’ richness (IndPSR), but only in females of *A. sylvaticus*.

Feeding behavior, social organization and hierarchy, parasite preferences, and differences in the parasite tolerances of the sexes could also explain the higher parasite load in females [18]. The same authors suggested that higher investment in resistance in circumstances of increased parasite risk could make females more tolerant of parasite diversity. Although it is prevalent, male-biased parasitism is not universal [67]. Female-biased parasitism has been reported in several rodent species from South Africa [68] and 16 neotropical bat species [69].

In contrast to BCI, the host’s spleen mass was not correlated with nematode species richness in our sample. Immune response was not dependent on parasite diversity. However, in *M. glareolus*, we found that the parasite load was higher in animals with a smaller spleen. The findings and conclusions of studies on the relationship between infection parameters and spleen size in other organisms are inconsistent. Some studies support the hypothesis that spleen size may reflect the intensity of immune response, and thus animals with higher infection levels should have a larger spleen. In the American mink, *Neovison vison* males with more parasite species had a larger spleen [49]. The same relationship was found in rats with higher numbers of *Toxocara canis* (Werner, 1782) larvae in the host’s muscles and brain [45]. On the other hand, Vicente et al. [70] found a negative relationship between red deer spleen size and infection with lungworm *Elaphostrongylus cervi*. Another negative correlation was described in geese by Shutler et al. [44].

Ponlet et al. [42] studied 12 rodent species from Thailand and found that spleen mass was not correlated with parasite species richness at the intraspecific level. However, at the interspecific level, they found that females may have evolved in response to gastrointestinal helminth pressure, unlike males. It seems that males, due to their higher physical activity, show physiological differences reflected in the size of the spleen. Immunocompetence measured by spleen mass seems less reliable in males [42]. A study on birds also demonstrated that the higher species’ richness of nematode parasites was correlated with an increased spleen size at the interspecific level. The spleen size differed between related bird species: those with a higher number of parasitic nematode species had a larger spleen [48].

It is obvious that parasites exert different levels of selective pressure, and in some cases, may provoke hosts to invest more in immune defense. We investigated intestinal helminths, ignoring other micro and macro parasites. The parasite species responsible for chronic infections such as nematodes tend to be involved in adaptive coevolutionary processes with their hosts [71,72]. Coexistence could allow the parasite to persist in limiting the number with minimal damage to the host. Less investment in immunity by host species in special circumstances allows certain parasite nematode species to increase their numbers in the organism of their host, as we obtained for *M. glareolus*. Immune function is costly and must be traded off against other life-history traits for better survival and reproductive success. Small rodent species usually have a short life span, and large investment in reproduction, so the allocation of energy to immunity does not take place when intestinal nematodes are considered.

## 5. Conclusions

In this study, we tested the relationships among the phenotypic characteristics (body mass, body condition index BCI, and spleen mass) of three small rodent species (*Apodemus sylvaticus*, *Apodemus flavicollis*, and *Myodes glareolus*) and its nematode parasites. A rich nematode fauna characterized the analyzed species since 15 nematode species were found. BCI showed a strong correlation with nematode species richness (IndPSR). This pointed out that animals in better condition could host more parasites while the parasite load (IndPL) does not follow this increase. Interestingly, in *A. flavicollis*, we found female-biased parasitism. Namely, females of the same sizes as the males, were parasitized with a higher number of nematode species. In contrast to BCI, the spleen mass did not show clear connections with the parasitological parameters.

## Figures and Tables

**Table 1:** The number of collected rodents across host species and localities.

Localities	*A. sylvaticus*	*A. flavicolis*	*M. glareolus*	Total
Zvezdarska šuma (Belgrade)	18	-	-	18
Ravnište (Jastrebac Mt.)	-	6	8	14
Vlasina	-	15	5	20
Babin zub (Stara planina Mt)	-	2	4	6
Petnica	-	12	4	12
Goč Mt.	-	5	-	9
Maljen Mt.	-	4	-	4
Ruski Krstur	-	5	-	5
Total	18	49	21	88

**Table 2:** Parasitological parameters for all nematode species in three small rodent species from localities in Serbia.

Parasites/Hosts	*Apodemus flavicollis*				*Apodemus sylvaticus*				*Myodes glareolus*			
	*n*	P%	MI	MA	*n*	P%	MI	MA	*n*	P%	MI	MA
*Aonchotheca annulosa*	120	10.2	24	2.45	4	11.1	2	0.22	25	14.3	8.33	1.19
*Aonchotheca murissylvatici*	-	-	-	-	-	-	-	-	70	9.5	35	3.33
*Aspiculuris tetraptera*	2	4.1	1	0.04	-	-	-	-	-	-	-	-
*Eucoleus* sp.	23	10.2	4.60	0.47	-	-	-	-	-	-	-	-
*Heligmosomoides* sp.	-	-	-	-	-	-	-	-	65	23.8	13	3.1
*Heligmosomum costellatum*	-	-	-	-	-	-	-	-	13	19.05	3.25	0.62
*Heligmosomoides glareoli*	1	2.0	1	0.02	-	-	-	-	56	66.7	4	2.67
*Heligmosomoides polygyrus*	357	69.4	10.50	7.29	10	33.3	1.67	0.56	2	4.7	2	0.10
*Heterakis spumosa*	1	2.0	1	0.02	-	-	-	-	-	-	-	-
*Mastophorus muris*	5	4.1	2.5	0.1	-	-	-	-	-	-	-	-
*Rictularia proni*	14	10.2	2.8	0.29	-	-	-	-	-	-	-	-
*Syphacia* sp.	1	2.0	1	0.02	10	16.7	3.33	0.56	-	-	-	-
*Syphacia frederici*	229	26.5	17.62	4.67	369	22.2	92.25	20.5	-	-	-	-
*Syphacia stroma*	702	28.6	50.14	14.33	-	-	-	-	-	-	-	-
*Trichuris muris*	532	28.6	38	10.86	-	-	-	-	1	4.8	1	0.05

*n*—number of parasites, P%—prevalence, MI—mean infection intensity, MA—mean abundance.

## Data Availability

The data supporting the findings of this study are available within the article.
